# Hsa-miR-557 Inhibits Osteosarcoma Growth Through Targeting KRAS

**DOI:** 10.3389/fgene.2021.789823

**Published:** 2022-01-11

**Authors:** Zhi Qiao, Jinfeng Li, Hongwei Kou, Xiangrong Chen, Deming Bao, Guowei Shang, Songfeng Chen, Yanhui Ji, Tian Cheng, Yisheng Wang, Hongjian Liu

**Affiliations:** Department of Orthopedics, The First Affiliated Hospital of Zhengzhou University, Zhengzhou, China

**Keywords:** hsa-miR-557, miRNA, osteosarcoma, KRAS, survival rate, animal model

## Abstract

**Objective:** Osteosarcoma is the most common malignancy in the skeletal system; studies showed an important role of miRNAs in tumorigenesis, indicating miRNAs as possible therapeutic molecules. This study found abnormal hsa-miR-557 expression levels in osteosarcoma and tried to explore the potential function and the mechanism.

**Methods:** Differential expression genes of osteosarcoma were analyzed using GSE28423 from the GEO database. Survival analysis of miRNAs was conducted with data obtained from the TARGET-OS database. STRING and miRDIP were used to predict target genes of hsa-miR-557; KRAS was then verified using dual-luciferase reporter assay. Expression of genes was detected by qPCR, and levels of proteins were detected by Western blot. The proliferation ability of cells was detected by CCK-8 and cell cycle analysis. Tumor formation assay in nude mice was used to detect the influence of osteosarcoma by hsa-miR-557 *in vivo*.

**Results:** Analysis from the GEO and TARGET databases found 12 miRNAs that are significantly related to the osteosarcoma prognosis, 7 downregulated (hsa-miR-140-3p, hsa-miR-564, hsa-miR-765, hsa-miR-1224-5p, hsa-miR-95, hsa-miR-940, and hsa-miR-557) and 5 upregulated (hsa-miR-362-3p, hsa-miR-149, hsa-miR-96, hsa-miR-744, and hsa-miR-769-5p). CCK-8 analysis and cell cycle analysis found that hsa-miR-557 could significantly inhibit the proliferation of osteosarcoma cells. The tumor formation assay in nude mice showed that tumor sizes and weights were inhibited by hsa-miR-557 transfection. Further studies also proved that hsa-miR-557 could target the 3′UTR of KRAS and modulate phosphorylation of downstream proteins.

**Conclusion:** This study showed that hsa-miR-557 could inhibit osteosarcoma growth both *in vivo* and *in vitro*, by modulating KRAS expression.

## Introduction

Osteosarcoma is one of the most common malignancies in the skeletal system, with an incidence of 2–3 million/year (Ritter and Bielack, 2010). Currently, around 70% of patients can be cured by surgical resection combined with chemotherapy. However, recurrent and metastatic osteosarcomas have a survival rate of only around 20%, which has remained unchanged for 30 years (Kansara et al., 2014). Thus, new therapies are needed for these osteosarcomas.

MicroRNAs (miRNAs) may be possible therapeutic molecules. Multiple studies showed the abnormal expression of miRNAs in malignancies, which indicated the role of miRNAs in tumorigenesis (Wang et al., 2019). Several *in vivo* studies showed the potential treatment effect of miRNAs in tumors (Wu et al., 2018). In osteosarcoma, molecules like miR-429 and miR-143-3p were found to be potential diagnostic and prognostic markers (Yang et al., 2020). Also, several molecules like miR-233-3p (Ji et al., 2018) and miR-1225-5p (Zhang et al., 2020) were found to be tumor suppressors of osteosarcoma. These results indicated potential diagnostic and treatment roles of miRNAs in osteosarcoma.

The Kirsten rat sarcoma virus (KRAS) gene belongs to the rat sarcoma virus (RAS) gene family, a group of genes that contribute to tumorigenesis. Publications have proved the association of KRAS with osteosarcoma, showing that KRAS could promote proliferation of osteosarcoma cells (Zhang et al., 2018a; Chen et al., 2019). KRAS was also proved to be targets of miRNAs. As previously reported, several miRNAs (miR-548-3p (Chen et al., 2019), miR-422a (Zhang et al., 2018a), and miR-217 (Zhang et al., 2015)) were shown to inhibit osteosarcoma by targeting KRAS. Thus, KRAS could be an important miRNA target that modulates the growth of osteosarcoma.

In this study, we found abnormal expression levels of hsa-miR-557 in osteosarcoma and tried to explore its potential function and mechanism.

## Material and Methods

### Bioinformatic Analysis

The Gene Expression Omnibus (GEO, https://www.ncbi.nlm.nih.gov/geo/) from the National Center for Biotechnology Information (NCBI) was searched, and data from the GSE28425 (a SuperSeries) were downloaded for miRNA expression analysis. GSE28425 includes GSE28423 (a non-coding RNA profile) and GSE28424 (an expression profile) of 19 osteosarcoma cell lines and 4 normal bones. MiRNA expression analysis was calculated using the R limma package (version 3.42.2). MiRNA expression data and clinical information of 89 osteosarcoma patients were downloaded from tumor alterations relevant for genomics-driven therapy (TARGET (https://xenabrowser.net/datapages/). Survival package (version 3.2–7) and survminer package (version 0.4.8) from R (version 3.5) were used for survival analysis. Psych package (version 2.0.9) was used for gene expression analysis, and the microRNA Data Integration Portal (miRDIP, http://ophid.utoronto.ca/mirDIP/) was used for target gene prediction. Protein–protein interaction (PPI) networks were constructed using STRING (https://string-db.org/).

### Cell Culture

One normal osteoblast cell line (HFOB1.19, ATCC, United States) and four osteosarcoma cell lines (U20S (ATCC, United States), MNNG/HOS (ATCC, United States), SAOS-2 (ATCC, United States), and MG63 (ATCC, United States)) were used. Cells were cultured in the complete culture medium (CCM: DMEM (Procell, Wuhan, China) +10% FBS (WISENT, Nanjing, China) +1% P/S (NCM Biotech, Suzhou, China)). Cells were cultured at 37°C with 5% CO_2_.

### qPCR Analysis

Cellular samples were put into 1 ml Trizol for 5 min and transferred into 1.5 ml Eppendorf for further use. Tissue samples were grinded in liquid nitrogen, treated with 1 ml Trizol, and then transferred into 1.5 ml Eppendorf. Total RNA was extracted with chloroform and cold isopropanol and washed with 75% ethanol 3 times. A reverse transcription kit (Genecopoeia, Guangzhou, China) was used to synthesize cDNAs according to the manufacturer’s instructions. BeyoFast™ SYBR Green qPCR Mix (Bio-Rad, Shanghai, China) was used for quantification of cDNAs, with the qTower 3.2G (Jena, Germany) qPCR instrument. The relative expression levels were analyzed using the 2^−ΔΔCt^ method. Triple wells were tested for each sample. Primers are shown in Table 1.

**TABLE 1:** Primers used in qPCR.

**GAPDH forward primer**	**ACA​GCC​TCA​AGA​TCA​TCA​GC**
GAPDH reverse primer	GGT​CAT​GAG​TCC​TTC​CAC​GAT
hsa-miR-557 RT primer	GTC​GTA​TCC​AGT​GCG​TGT​CGT​GGA​GTC​GGC​AAT​TGC​ACT​GGA​TAC​GAC​AGA​CAA
hsa-miR-557 forward primer	GTTTGCACGGGTGGGCC
hsa-miR-557 reverse primer	CAGTGCGTGTCGTGGA
KRAS forward primer	GGA​CTG​GGG​AGG​GCT​TTC​T
KRAS reverse primer	GCC​TGT​TTT​GTG​TCT​ACT​GTT​CT

### Western Blot

Cell samples were lysed with 2×SDS lysis buffer (Beyotime Biotechnology, Shanghai, China). Tissues were grinded in liquid nitrogen and treated with RIPA (Beyotime Biotechnology, Shanghai, China). Quantification of proteins was performed using BCA (bicinchoninic acid assay). Protein samples were isolated in SDS-PAGE gel by electrophoresis and transferred to a PVDF membrane. GAPDH mouse (proteintech, Wuhan, China), *p*-ERK1/2 (Santa Cruz, United States), ERK1/2 (proteintech, Wuhan, China), *p*-JNK (proteintech, Wuhan, China), JNK (proteintech, Wuhan, China), KRAS (proteintech, Wuhan, China), anti-mouse IgG (H + L) (CST, United States), and anti-rabbit IgG (H + L) (CST, United States) were used to incubate the PVDF membrane according to the detecting proteins. Results were read *via* chemiluminescence (Tanon 5200 multi, Shanghai, China) using an ECL kit (Beyotime Biotechnology, Shanghai, China).

### CCK-8 Assay

The cell counting kit-8 (CCK-8) assay was used to assess cell viability. Manufactured cells were cultured in a 96-well plate at a density of 5 × 10^3^ cells per well, with 5% CO_2_ at 37°C. After 0, 24, 48, and 72 h, 10 μL CCK-8 (Sigma, United States) was mixed and cultured for 2h at 37°C. The optical density (OD) values at 450 nm were recorded for analysis. Three repetitions were measured for each sample.

### Cell Cycle Analysis

Manufactured cells were suspended with trypsin (NCM Biotech, Suzhou, China), washed twice with cold PBS, and fixed with 75% ethanol at 4°C for 24 h. After fixation, cells were washed twice with PBS and incubated in RNase A (Beyotime Biotechnology, Shanghai, China) and propidium iodide (PI, Beyotime Biotechnology, Shanghai, China) for 1h. The percentage of cells in different cell cycles was tested using a flow cytometer (ACEA NovoCyte D2040R, United States). The proliferation index (PI) was used for analysis. PI=(S + G2)/(G1+S + G2). Three repetitions were measured for each group.

### Plasmid Construction

The PLVX-IRES-ZsGreen1 (ORL-BIO, Hunan, China) plasmid was used as a gene vector, and the psiCHECK-2 (ORL-BIO, Hunan, China) plasmid was used for dual-luciferase reporter assay. The coding sequence (CDS) of KRAS was downloaded from the NCBI, and primers were designed using CE DesignⅤ (version 1.04). cDNAs were replicated by PCR and collected using an Agarose Gel Extraction kit (Omega, Georgia, United States). The QuickCut™ XhoI and the QuickCut™ EcoR I were used to cut plasmids. Plasmids and cDNAs were connected in Seamless Master Mix (Beyotime Biotechnology, Shanghai, China). Gene vectors were transformed into the competent DH5α *Escherichia coli*, cultured in the amp culture medium. Positive bacteria were selected. Plasmids were extracted and sent for sequencing. Sequences used are seen in Table 2.

**TABLE 2:** Sequences used in plasmid construction.

**Hsa-miR-557**	**GUU​UGC​ACG​GGU​GGG​CCU​UGU​CU**
Hsa-miR-557 inhibitor	AGA​CAA​GGC​CCA​CCC​GUG​CAA​AC
NC miRNA	UCA​CAA​CCU​CCU​AGA​AAG​AGU​AGA
NC miRNA inhibitor	UCU​ACU​CUU​UCU​AGG​AGG​UUG​UGA
Wt-KRAS forward primer	TCG​AGA​GGC​TCT​ATT​TAA​CTG​AGT​CAC​ACT​GCA​TAG​GAA​TTT​AGG​TAC​CGC
Wt-KRAS reverse primer	GGC​CGC​GGT​ACC​TAA​ATT​CCT​ATG​CAG​TGT​GAC​TCA​GTT​AAA​TAG​AGC​CTC
Mut-KRAS forward primer	TCG​AGA​GGC​TCT​ATT​TAA​CTG​ATA​GCA​GTT​GCA​TAG​GAA​TTT​AGG​TAC​CGC
Mut-KRAS reverse primer	GGC​CGC​GGT​ACC​TAA​ATT​CCT​ATG​CAA​CTG​CTA​TCA​GTT​AAA​TAG​AGC​CTC

### Lentivirus Packaging

For lentivirus packaging, 293 T cells were used. Cells were cultured until 70% coverage. The culture medium was renewed with serum-free DMEM 2 h before transfection. Cells were cocultured with pHelper1, pHelper2, the constructed plasmid (1:1.5:2), and the lipofectamine 2000 for transfection. The cells were washed with PBS after 8 h, the culture medium was renewed, and cells were cultured for 48 h. A fluorescence microscope was used to observe the transfection efficiency.

### Tumor Formation in Nude Mice

The animal experiment was proven by the research and clinical trial ethics committee of Zhengzhou University (No.2021-KY-0035–001). Twelve nude mice were separated into two groups, the experimental group and the control group. MG63 cells infected with lentivirus encoding has-miR-557 or empty vectors were harvested and resuspended in the complete culture medium. Cellular density was adjusted to 2 × 10^7^/ml with the complete culture medium and matrigel (3:1). Each mouse was subcutaneously injected with 2×10^6^ cells in 0.1 ml. Mice were raised at 24°C, humidity 30–40%, and 12 h light per day, with enough food and water supply. Tumors were sized from the eighth day (when visible) and sized every 5 days afterward. The mice were terminated on the 28th day, and tumors were collected for further analysis.

### Dual-Luciferase Reporter Assay

The manufactured psiCHECK-2 plasmid was transfected into 293T cell line (ATCC, United States) by Lipo 2000 (Thermo Fisher Scientific, United States), together with the hsa-miR-557 or hsa-miR-557 inhibitor or the negative control (NC), accordingly. Dual-luciferase reporter assays (Beyotime Biotechnology, Shanghai, China) were performed according to the manufacturer’s instruction. Relative light unit (RLU) of firefly and Rinilla luciferase were recorded.

### Statistical Analysis

Statistical analysis was performed using GraphPad Prism 8 (GraphPad Software, La Jolla, CA) and R (version 3.5). Graphs were created using GraphPad Prism 8 (GraphPad Software, La Jolla, CA). The Student’s *t*-test and Mann–Whitney test were used in comparisons between two groups, ANOVA was used for comparisons between three or more groups. Pearson’s correlation was used for correlation analysis. *p* < 0.05 was considered as statistically significant.

## Results

### Hsa-miR-557 Positively Correlate With the Survival Rate of Osteosarcoma Patients

**FIGURE 1:**
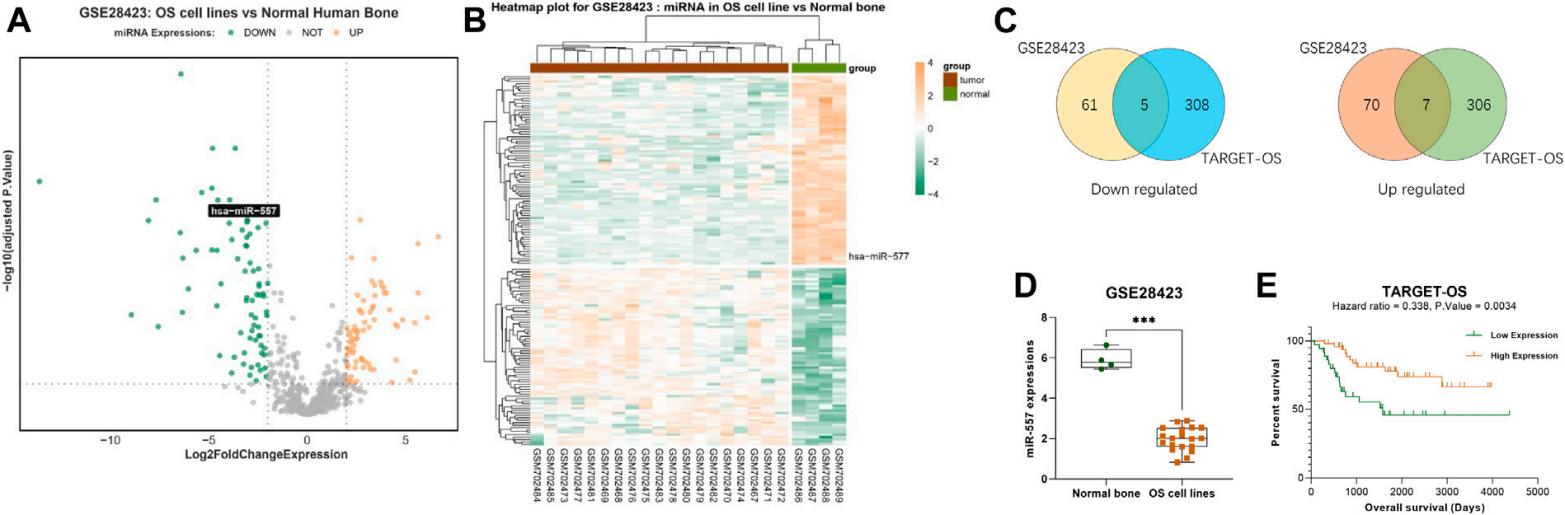
Hsa-miR-557 positively correlate with the survival rate of osteosarcoma patients: **(A,B)** GSE28425 dataset from the GEO showed 74 upregulated miRNAs (log_2_foldchange >2, adjusted *p*. value <0.05) and 79 downregulated miRNAs (log_2_foldchange < -2, adjusted *p*. value <0.05). **(C)** Combining data from GSE28425 with 89 OS patient samples from TARGET, 7 miRNAs were downregulated in the GSE28425 dataset and lowly expressed in the TARGET database (hsa-miR-140-3p, hsa-miR-564, hsa-miR-765, hsa-miR-1224-5p, hsa-miR-95, hsa-miR-940, and hsa-miR-557), and 5 miRNAs (hsa-miR-362-3p, hsa-miR-149, hsa-miR-96, hsa-miR-744, and hsa-miR-769-5p) were upregulated in the GSE28425 dataset and highly expressed in the TARGET database. **(D)** Significant low levels of hsa-miR-557 were found in osteosarcoma cell lines from GSE28425. **(E)** Data from TARGET-OS showed that hsa-miR-557 positively correlate with the survival rate of osteosarcoma patients (hazard ratio <1, *p*. value < 0.01).

To investigate the differently expressed miRNAs in osteosarcoma, we searched the GEO database (https://www.ncbi.nlm.nih.gov/geo/) from the NCBI and found the GSE28425 (Namlos et al., 2012) dataset. Within this dataset, 4 normal human bone tissues and 19 osteosarcoma cell lines (143B, HAL, HOS, IOR-MOS, IOR-OS10, IOR-OS14, IOS-OS15, IOS-OS18, IOS-OS9, KPD, MG-63, MHM, MNHG-HOS, OHS, SaOS-2, U-2OS, and ZK-58) were analyzed. 153 miRNAs were found to express differently, including 74 upregulated miRNAs (log_2_foldchange >2, adjusted p. value <0.05) and 79 downregulated miRNAs (log_2_foldchange < -2, adjusted *p*. value <0.05) (Figures 1A,B).

Data of 89 osteosarcoma patients’ samples from the TARGET database were also analyzed, including 58,389 genes. 314 miRNAs were found that may influence the survival rate of OS patients (hazard ratio >1 or <1, *p*. value <0.05). Taking the intersection with differently expressed miRNAs from the GSE28425 dataset, 12 miRNAs were found that correlate with poor prognosis: 7 miRNAs were downregulated in the GSE28425 dataset and lowly expressed in the TARGET database (hsa-miR-140-3p, hsa-miR-564, hsa-miR-765, hsa-miR-1224-5p, hsa-miR-95, hsa-miR-940, and hsa-miR-557), and 5 miRNAs (hsa-miR-362-3p, hsa-miR-149, hsa-miR-96, hsa-miR-744, and hsa-miR-769-5p) were upregulated in the GSE28425 dataset and highly expressed in the TARGET database (Figure 1C). Among these genes, hsa-miR-557 was found to be significantly downregulated (log2foldchange = −3.958, p. value = 1.083e-10, adjusted *p*. value = 4.808e-09) (Figure 1D); at the same time, higher expression of hsa-miR-557 positively correlates with a better survival rate of osteosarcoma patients (hazard ratio <1, *p*. value < 0.01) (Figure 1E).

### Hsa-miR-557 Inhibits Proliferation of Osteosarcoma Cells *In Vitro*

**FIGURE 2:**
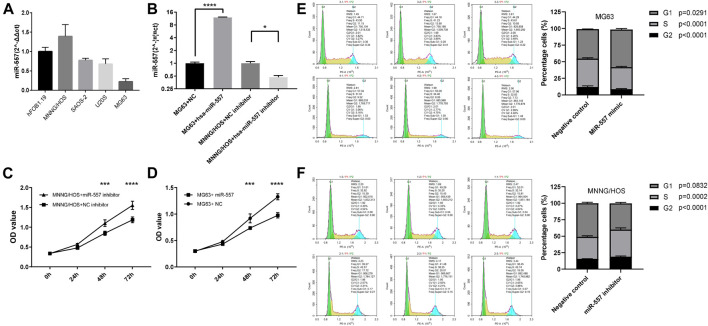
Hsa-miR-557 inhibits the proliferation of osteosarcoma cells *in vitro*. **(A)** Hsa-miR-557 expression in osteosarcoma cell lines (MNNG/HOS, SAOS-2, U20S, and MG63) and normal osteoblasts (hFOB1.19). **(B)** Successfully elevated hsa-miR-557 expression levels by hsa-miR-557 transfection in MG63 and inhibited hsa-miR-557 expression levels by hsa-miR-557 inhibitor transfection in MNNG/HOS. **(C,D)** CCK-8 assay showed that Hsa-miR-557 inhibited MG63 cell proliferation, and hsa-miR-557 inhibitor could promote MNNG/HOS cell proliferation. **(E,F)** Cell cycle analysis showed that comparing with the NC, hsa-miR-557 inhibited the proliferation of MG63 cells (*p* = 0.0008, t = 8.999), and inhibition of hsa-miR-557 promoted MNNG/HOS proliferation (*p* = 0.0001, t = 14.62) (*p < 0.05, ***p < 0.001, ****p < 0.0001).

Expression levels of hsa-miR-557 in different osteosarcoma cell lines (MNNG/HOS, SAOS-2, U20S, and MG63) and normal osteoblasts (hFOB1.19) were analyzed. Results showed that MNNG/HOS cells have the highest level of hsa-miR-557 expression, while MG63 cells have the lowest level of hsa-miR-557 expression (Figure 2A). Thus, MNNG/HOS and MG63 were selected to analyze the function of hsa-miR-557 on osteosarcoma *in vitro*. MNNG/HOS cells were transfected with the hsa-miR-557 inhibitor to specifically knock down hsa-miR-557, and MG63 cells were transfected with hsa-miR-557 to upregulate has-miR-557 expression. Results of qPCR showed that hsa-miR-557 levels were increased in MG63 cells and decreased in MNNG/HOS cells (MG63: *p* < 0.0001, q = 111.6; MNNG/HOS: *p* = 0.02, q = 5.458) (Figure 2B). To test the effect of hsa-miR-557 on cellular proliferation, CCK-8 assay and cell cycle analysis were performed. CCK-8 assay showed that the proliferation of MG63 cells was inhibited by hsa-miR-557 compared with the NC (48 h: *p* = 0.0003, t = 5.205; 72 h: *p* < 0.0001, q = 9.928) (Figure 2C), and the activity of MNNG/HOS cells was promoted by the hsa-miR-557 inhibitor, compared with the NC (48 h: *p* = 0.0003, t = 5.305; 72 h: *p* < 0.0001, t = 7.603) (Figure 2D). Cell cycle analysis also showed that comparing with the NC, inhibition of hsa-miR-557 promoted MNNG/HOS proliferation (*p* = 0.0001, t = 14.62), while hsa-miR-557 led to an inhibition of MG63 proliferation (*p* = 0.0008, t = 8.999) (Figures 2E,F).

### Hsa-miR-557 Inhibit Growth of Osteosarcoma *In Vivo*

**FIGURE 3:**
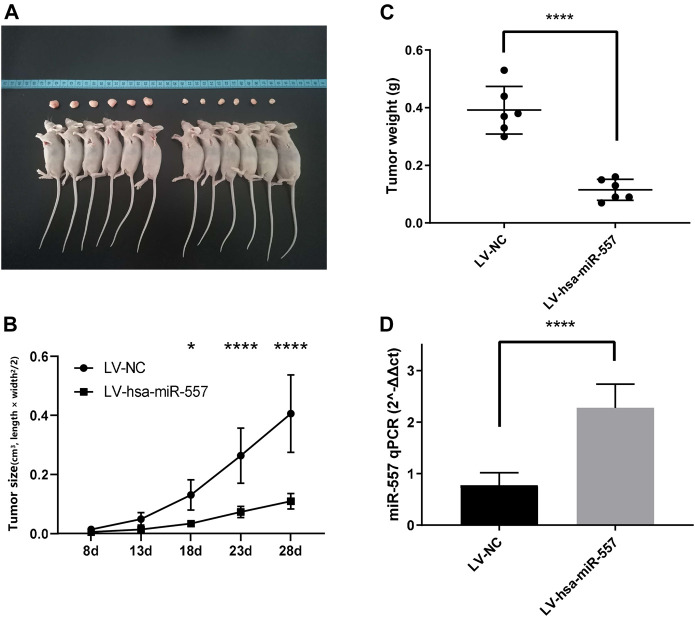
Hsa-miR-557 inhibited the growth of osteosarcoma *in vivo*. MG63 cells transfected with hsa-miR-557 or the negative control (NC) by lentivirus (LV) were injected into BALB/c nude mice subcutaneously. **(A)** Tumor sizes from the hsa-miR-557 group and the negative control group after 28 days. **(B)** Tumor volume was measured every 5 days from the 8th day. **(C)** Significant lighter tumor weight was tested in the hsa-miR-557 group. **(D)** Higher levels of hsa-miR-557 expression in the hsa-miR-557 overexpression group were verified (*p < 0.05, ****p < 0.0001).

To test if hsa-miR-557 could inhibit the growth of osteosarcoma *in vivo*, nude mice were used to establish a xenograft mouse model. Results showed that hsa-miR-557 could inhibit the growth of osteosarcoma, as the tumor size and tumor weight were significantly reduced when has-miR-557 was upregulated (Figures 3A–C). Expression levels of hsa-miR-557 in tumor tissue samples were tested by qPCR. The tumor tissue transfected with hsa-miR-557 showed significantly high levels of hsa-miR-557 expression compared with the NC group (*p* < 0.0001, t = 7.083) (Figure 3D).

### Hsa-miR-557 Targets KRAS and Negatively Regulates KRAS Expression

**FIGURE 4:**
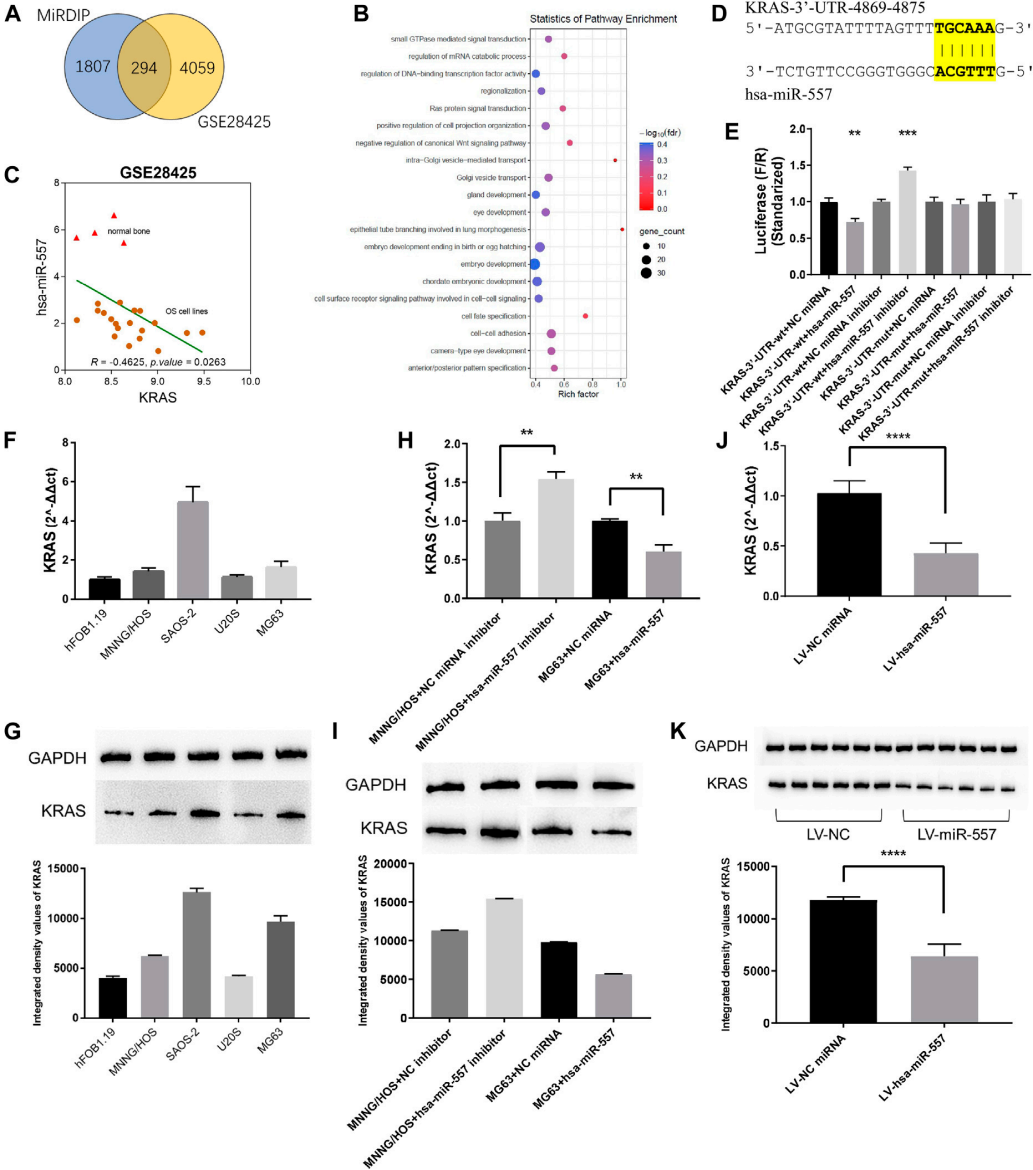
Hsa-miR-557 inhibited the growth of osteosarcoma through KRAS. **(A)** Combing the GSE28425 dataset with the miRDIP database, 294 hsa-miR-557 target genes were found to be significantly downregulated. **(B)** Pathway enrichment using STRING showed that the RAS signaling pathway was significantly involved. **(C)** Data from GSE28425 showed a significant negative correlation between KRAS and hsa-miR-557. **(D)** Simulation of hsa-miR-557 target sequence of KRAS 3′UTR. **(E)** Dual-luciferase reporter assay showed that lower levels of F/R were detected in the KRAS-3′U TR-wt + hsa-miR-557 group, and higher levels of F/R were detected in the KRAS-3′UTR-wt + hsa-miR-557 inhibitor group. **(F,G)** Expression level of KRAS in osteosarcoma cell lines (MNNG/HOS, SAOS-2, U20S, and MG63) and normal osteoblasts (hFOB1.19). **(H,I)** Levels of KRAS were inhibited by hsa-miR-557 in MG63 cells and promoted by hsa-miR-557 inhibitor in MNNG/HOS cells. **(J,K)** In the nude mice model, lower levels of KRAS expression were tested in the hsa-miR-557 vector group (**p < 0.01, ****p < 0.0001).

MiRNA was considered to play a critical role in modulating gene expressions through binding to the 3′UTR of its target gene, thus leading to a degradation of the target gene. To disclose the potential mechanism of hsa-miR-557 in osteosarcoma progression, the GSE28425 dataset was used to analyze genes related to has-miR-557. 4,353 negatively expressed genes were found. Furthermore, 2,100 potential targets of has-miR-557 were obtained from the miRDIP (Figure 4A). Genes were then subjected to performing PPI networks using STRING. Results showed that the Ras protein signaling transduction pathway was involved in hsa-miR-557 modulation (Figure 4B), and KRAS was found to be a target gene of hsa-miR-557, while its expression was found to correlate significantly with hsa-miR-557 levels (Figure 4C).

To further verify the potential mechanism of hsa-miR-557 in modulating osteosarcoma cell proliferation, dual-luciferase reporter assays were performed. Gene sequence of KRAS was downloaded from the NCBI (https://www.ncbi.nlm.nih.gov/gene), Inctar (http://www.cuilab.cn/lnctar) was used to predict the potential target site of hsa-miR-557 seed sequence (Figure 4D). Wild-type or mutant KRAS 3′UTR (KRAS-3′UTR-wt and KRAS-3′UTR-mut) was integrated into the psiCHECK-2 plasma. Hsa-miR-557, hsa-miR-557 inhibitor, NC miRNA, and NC miRNA inhibitor were constructed and transfected into the 293T cell line together with the KRAS-3′UTR-wt or the KRAS-3′UTR-mut, respectively. Sequences are seen in Table 2. Results showed that compared with KRAS-3′UTR-wt + NC miRNA, levels of F/R (Firefly luciferase/Renilla luciferase) in the KRAS-3′UTR-wt + hsa-miR-557 group were significantly lower (*p* = 0.0031, t = 6.366), while the level of F/R in the KRAS-3′UTR-wt + hsa-miR-557 inhibitor group was significantly higher (*p* = 0.0006, t = 9.820) (Figure 4E). These results indicated that hsa-miR-557 could target the 3′UTR of KRAS, inhibiting the expression of KRAS.

To verify the correlation of hsa-miR-557 with KRAS, levels of KRAS in osteosarcoma and osteoblast cell lines were tested. KRAS expression levels were higher in osteosarcoma cell lines than the osteoblast cell line hFOB1.19, tested by qPCR (*p* < 0.0001) and Western blot (*p* < 0.0001) (Figures 4F,G). Then, MNNG/HOS cells transfected with the hsa-miR-557 inhibitor and NC miRNA inhibitor, as well as MG63 cells transfected with hsa-miR-557 and NC miRNA, were constructed. Results showed that the overexpression of hsa-miR-557 reduced both the protein and mRNA levels of KRAS, while the inhibition of hsa-miR-557 resulted in an upregulation of KRAS levels (Figures 4H,I). Similar results were also found *in vivo* (Figure 4J,K).

### Hsa-miR-557 Inhibit Growth of Osteosarcoma Through Targeting KRAS and the Downstream Molecules

**FIGURE 5:**
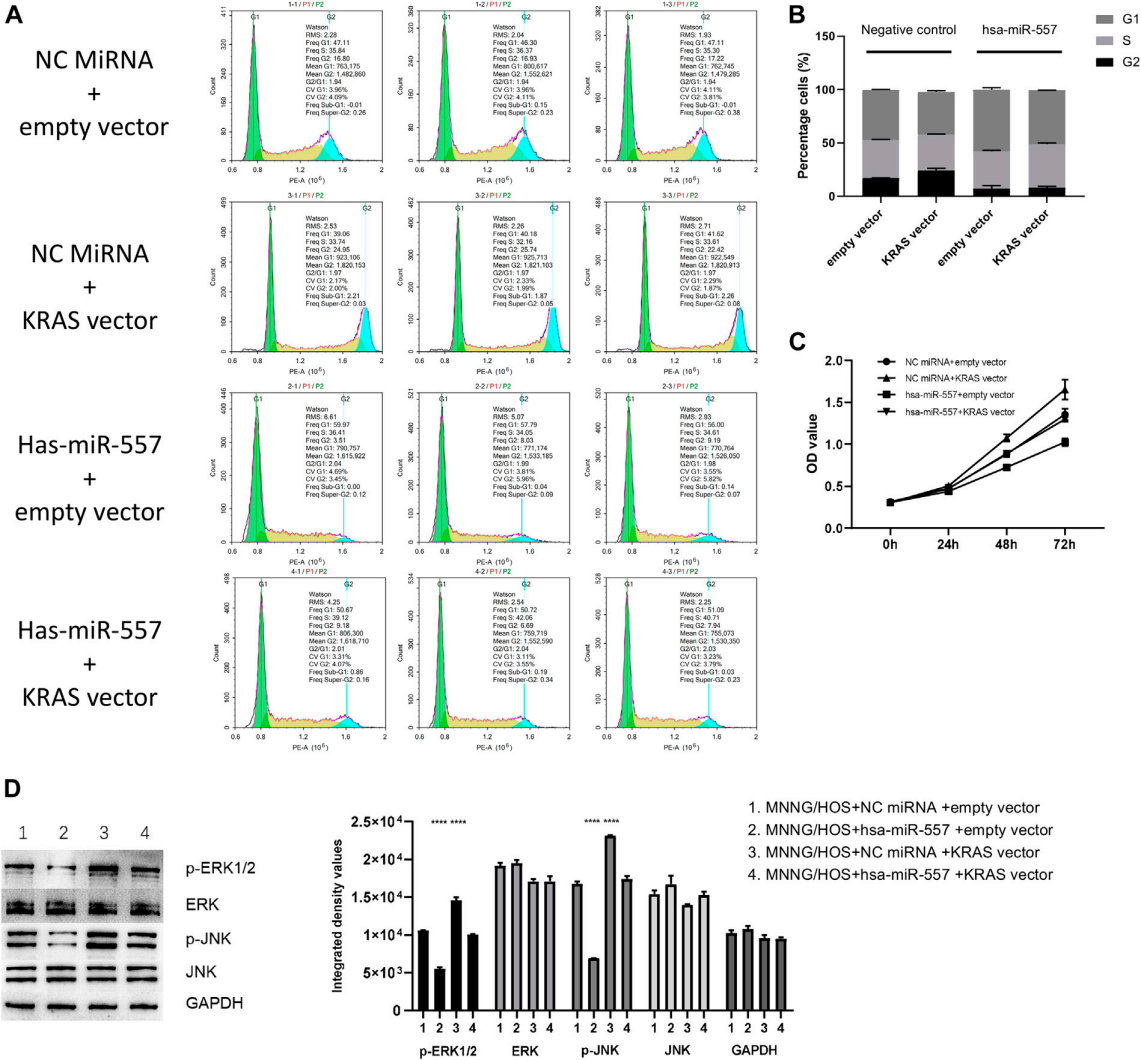
Hsa-miR-557 inhibits the growth of osteosarcoma through targeting KRAS and its downstream molecules. **(A,B)** Cell cycle analysis showed that hsa-miR-557 could inhibit the proliferation of osteosarcoma cells through KRAS (NC miRNA + empty vector vs NC miRNA + KRAS vector: *p* = 0.0021, t = 7.114, NC miRNA + empty vector vs hsa-miR-557 + empty vector: *p* = 0.0007, t = 9.457, hsa-miR-557 + KRAS vector vs hsa-miR-557 + empty vector: *p* = 0.0038, t = 6.039). **(C)** CCK-8 showed that KRAS could alleviate the inhibition effect of hsa-miR-557 on osteosarcoma (NC miRNA + empty vector vs hsa-miR-557 + empty vector: *p* (48 h) = 0.0003, q (48 h) = 6.522, *p* (72 h) < 0.0001, q (72 h) = 13.84); NC miRNA + empty vector vs NC miRNA + KRAS: *p* (48 h) < 0.0001, q (48 h) = 8.080, *p* (72 h) < 0.0001, q (72 h) = 12.380; NC miRNA + KRAS vector vs hsa-miR-557 + KRAS vector, *p* (48 h) < 0.0001, q (48 h) = 7.815, *p* (72 h) < 0.0001, q (72 h) = 14.72). **(D)** Phosphorylation of ERK1/2 and JNK was promoted by KRAS with NC miRNA and inhibited by hsa-miR-557 mimic (****p < 0.0001).

To certify that hsa-miR-557 could inhibit the proliferation of osteosarcoma, the psiCHECK-2 plasma integrated with KRAS 3′UTR (KRAS vector), the psiCHECK-2 plasma without KRAS 3′UTR (empty vector) were constructed. Hsa-miR-557 and NC miRNA were constructed according to Table 2. CCK-8 and cell cycle analysis were performed. Results showed that compared with the NC miRNA + empty vector group, KRAS could promote the proliferation of osteosarcoma cells (NC miRNA + KRAS vector, *p* = 0.0021, t = 7.114) and hsa-miR-557 could inhibit the proliferation of osteosarcoma cells (hsa-miR-557 + empty vector, *p* = 0.0007, t = 9.457), while the transfection of the KRAS vector could alleviate the inhibition effect of hsa-miR-557 (hsa-miR-557 + KRAS vector vs hsa-miR-557 + empty vector, *p* = 0.0038, t = 6.039) (Figures 5A,B). Results of CCK-8 also showed that compared with the NC miRNA + empty vector group, hsa-miR-557 could inhibit the proliferation of osteosarcoma cells at 48 h (*p* = 0.0003, q = 6.522) and 72 h (*p* < 0.0001, q = 13.84), KRAS could promote the proliferation of osteosarcoma cells (48h: *p* < 0.0001, q = 8.080; 72 h: *p* < 0.0001, q = 12.380), while the transfection of hsa-miR-557 could inhibit the effect of KRAS (NC miRNA + KRAS vector vs hsa-miR-557 + KRAS vector, 48 h: *p* < 0.0001, q = 7.815; 72 h: *p* < 0.0001, q = 14.72) (Figure 5C). These results proved the inhibition role of hsa-miR-557 in osteosarcoma proliferation through KRAS.

In the test of downstream proteins, four groups were built: ①MG63 + NC miRNA + empty vector, ②MG63 + hsa-miR-557 + empty vector, ③MG63 + NC miRNA + KRAS vector, and ④MG63+ hsa-miR-557 + KRAS vector. Downstream proteins (*p*-ERK1/2, ERK, *p*-JNK, JNK) were then tested by Western blot. Results showed that higher levels of *p*-ERK1/2 and *p*-JNK were found in the MG63 + NC miRNA + KRAS group, while lower levels of *p*-ERK1/2 and *p*-JNK were seen in the MG63+hsa-miR-557 + empty vector group (Figure 5D). Inhibition of *p*-ERK1/2 and *p*-JNK could be alleviated by KRAS. Results indicated that KRAS could promote the phosphorylation of ERK1/2 and JNK, while hsa-miR-557 could inhibit this process.

## Discussion

Hsa-miR-557 was recently noticed by researchers, as it has abnormal expression levels in several malignancies (Katayama et al., 2012; Jiang et al., 2014). We also noticed the abnormality of hsa-miR-557 expression in osteosarcoma and tried to find its function and the mechanism in modulating osteosarcoma. In this study, we found the following results: 1. hsa-miR-557 positively correlates with the survival rate of osteosarcoma patients. 2. hsa-miR-557 inhibits the proliferation of osteosarcoma cells *in vitro*. 3. hsa-miR-557 inhibits the growth of osteosarcoma *in vivo*. 4. hsa-miR-557 inhibits the growth of osteosarcoma through KRAS and its downstream molecules.

Several miRNAs were reported to have potential in osteosarcoma treatment. MiR-328-3p was reported to increase the radiosensitivity of osteosarcoma cells, inhibit the proliferation, and promote apoptosis through H2AX (Yang et al., 2018). Dai et al. reported that miR-513a-5p could enhance the radiosensitivity of OS, by negatively regulating APE1 (Dai et al., 2018). Mir-92a could inhibit the growth and the migration of osteosarcoma cells through targeting Notch1 (Liu et al., 2018). Other publications showed miR-29, miR-590, and miR-140-5p also inhibit proliferation and promote apoptosis of osteosarcoma cells (Wei et al., 2016; Xu et al., 2018; Long and Lin, 2019). On the other hand, several miRNAs were overexpressed in osteosarcoma, assisting in tumorigenesis. Zhang et al. found that miR-19a-3p could inhibit PTEN, thus promoting the proliferation of osteosarcoma cells (Zhang et al., 2019). Mir-21 was found to be overexpressed in osteosarcoma patients, and miR-21 knockout could reduce the invasion of the MG63 osteosarcoma cell line (Zhang et al., 2019).

Hsa-miR-557 was previously reported to be related to tumorigenesis and the survival rate. Abnormal hsa-miR-557 expression was found in patients with hepatocellular carcinoma (Katayama et al., 2012), gastric cancer (Jiang et al., 2014), pancreatic ductal adenocarcinoma (Zhang et al., 2018b), and lung cancers (Qiu et al., 2017). In addition, hsa-miR-557 was reported to be a tumor suppressor. Qiu et al. reported that miR-557 could inhibit lung cancer by negatively regulating lymphocyte enhancement factor 1. Razaviyan et al. found that mir-557 could inhibit S6K1 in breast cancer (Razaviyan et al., 2018). MiR-557 was also found to suppress pancreatic cancer cells through miR-557/SLC7A11/PI3K/AKT (Zhang et al., 2021). In this study, we found that hsa-miR-557 was positively correlated with the survival rate of osteosarcoma patients. Through *in vivo* and *in vitro* study, hsa-miR-557 was proved to be a tumor suppressor of osteosarcoma.

In a recent study, Wang et al. noticed the suppressive effect of hsa-miR-557 on osteosarcoma (Wang et al., 2021). Their study found that hsa-miR-557 could inhibit the malignant behavior of osteosarcoma by reducing HOXB9, which can deactivate the epithelial-mesenchymal transition (EMT) process. In our study, KRAS was found to be a target of hsa-miR-557, inhibiting the proliferation of osteosarcoma cells. As known, each miRNA could have multiple targets, which modulate cellular behaviors in the same or different directions. Combining these two studies, more evidence was offered, proving that hsa-miR-557 plays an inhibiting role in osteosarcoma. Therefore, hsa-miR-557 could be a promising treatment molecule in osteosarcoma.

MiRNAs inhibit translation or degrade mRNA molecules through binding to the complementary mRNA sequence (Rupaimoole and Slack, 2017). Several tumor-related genes were reported previously as targets of has-miR-557. Yang et al. found EGFR to be one of the has-miR-557 targets in inhibiting the proliferation and invasion of pancreatic cancer (Yang et al., 2019). Qiu et al. found that miR-557 could suppress lung cancer by regulating LEF1 (Qiu et al., 2017). In this study, KRAS was proved to be one of the hsa-miR-557 target molecules. KRAS is a famous mutated oncogene seen in a variety of tumors, among them is osteosarcoma. The activation of KRAS could switch on downstream pathways causing cell growth, differentiation, and influence survival (Uprety and Adjei, 2020). At the same time, the disturbance of KRAS suppressed tumor growth (Zhang et al., 2018a; Chen et al., 2019). ERK and JNK are important RAS downstream molecules, controlling cell growth and survival. Phosphorylation levels of ERK and JNK are associated with poor prognosis of osteosarcoma patients (Li et al., 2016; Czarnecka et al., 2020). The inhibition of ERK and JNK phosphorylation by hsa-miR-557 indicates its potential in treating osteosarcoma. Additionally, ERK and JNK are downstream molecules of KRAS in tumorigenesis (Cellurale et al., 2011; Samatar and Poulikakos, 2014). Modulation of KRAS downstream molecules (ERK and JNK) by hsa-miR-557 reconfirmed the relationship between them.

In conclusion, the present study demonstrated that hsa-miR-557 could inhibit the proliferation of osteosarcoma cells through modulating KRAS expression.

## Data Availability

The datasets presented in this study can be found in online repositories. The names of the repository/repositories and accession number(s) can be found in the article/Supplementary Material.
